# Identification and characterization of mushroom body neurons that regulate fat storage in *Drosophila*

**DOI:** 10.1186/s13064-018-0116-7

**Published:** 2018-08-13

**Authors:** Bader Al-Anzi, Kai Zinn

**Affiliations:** 1 Food & Nutrition Program, Environment & Life Sciences Research Center, Kuwait Institute for Scientific Research, P.O. Box 24885, 13109 Kuwait City, Kuwait; 2 Division of Biology and Biological Engineering, California Institute of Technology, Pasadena, CA 91125 USA

## Abstract

**Background:**

In an earlier study, we identified two neuronal populations, c673a and Fru-GAL4, that regulate fat storage in fruit flies. Both populations partially overlap with a structure in the insect brain known as the mushroom body (MB), which plays a critical role in memory formation. This overlap prompted us to examine whether the MB is also involved in fat storage homeostasis.

**Methods:**

Using a variety of transgenic agents, we selectively manipulated the neural activity of different portions of the MB and associated neurons to decipher their roles in fat storage regulation.

**Results:**

Our data show that silencing of MB neurons that project into the α’β’ lobes decreases de novo fatty acid synthesis and causes leanness, while sustained hyperactivation of the same neurons causes overfeeding and produces obesity. The α’β’ neurons oppose and dominate the fat regulating functions of the c673a and Fru-GAL4 neurons. We also show that MB neurons that project into the γ lobe also regulate fat storage, probably because they are a subset of the Fru neurons. We were able to identify input and output neurons whose activity affects fat storage, feeding, and metabolism. The activity of cholinergic output neurons that innervating the β’2 compartment (MBON-β’2mp and MBON-γ5β’2a) regulates food consumption, while glutamatergic output neurons innervating α’ compartments (MBON-γ2α’1 and MBON-α’2) control fat metabolism.

**Conclusions:**

We identified a new fat storage regulating center, the α’β’ lobes of the MB. We also delineated the neuronal circuits involved in the actions of the α’β’ lobes, and showed that food intake and fat metabolism are controlled by separate sets of postsynaptic neurons that are segregated into different output pathways.

**Electronic supplementary material:**

The online version of this article (10.1186/s13064-018-0116-7) contains supplementary material, which is available to authorized users.

## Background

Regulation of fat storage and metabolism by the brain requires collaboration among many types of neurons. In mammals, body weight is controlled by specific brain regions, including subdivisions of the hypothalamus [11, 48]. These hypothalamic nuclei contain a variety of neuronal types that can have both behavioral (e.g.*,* feeding and physical activity) and metabolic outputs (e.g.*,* controlling basal metabolic rate, altering rates of de novo fatty acid synthesis) that must be coordinated to ensure that fat content is set at the appropriate levels [11, 12, 53].

In *Drosophila*, genetic screens for alterations in feeding behavior and metabolism have identified many genes. These include components of the insulin and serotonin pathways, which are known to regulate body weight in mammals [16, 25–27, 36, 39, 44]. *Drosophila* is also an excellent model in which to examine how neuronal activity influences behavior and physiology. This is largely due to the availability of thousands of neuron-specific GAL4 ‘driver’ lines, with which it is possible to turn activity up or down in specific and localized populations of neurons [23, 35].

**Fig. 1:**
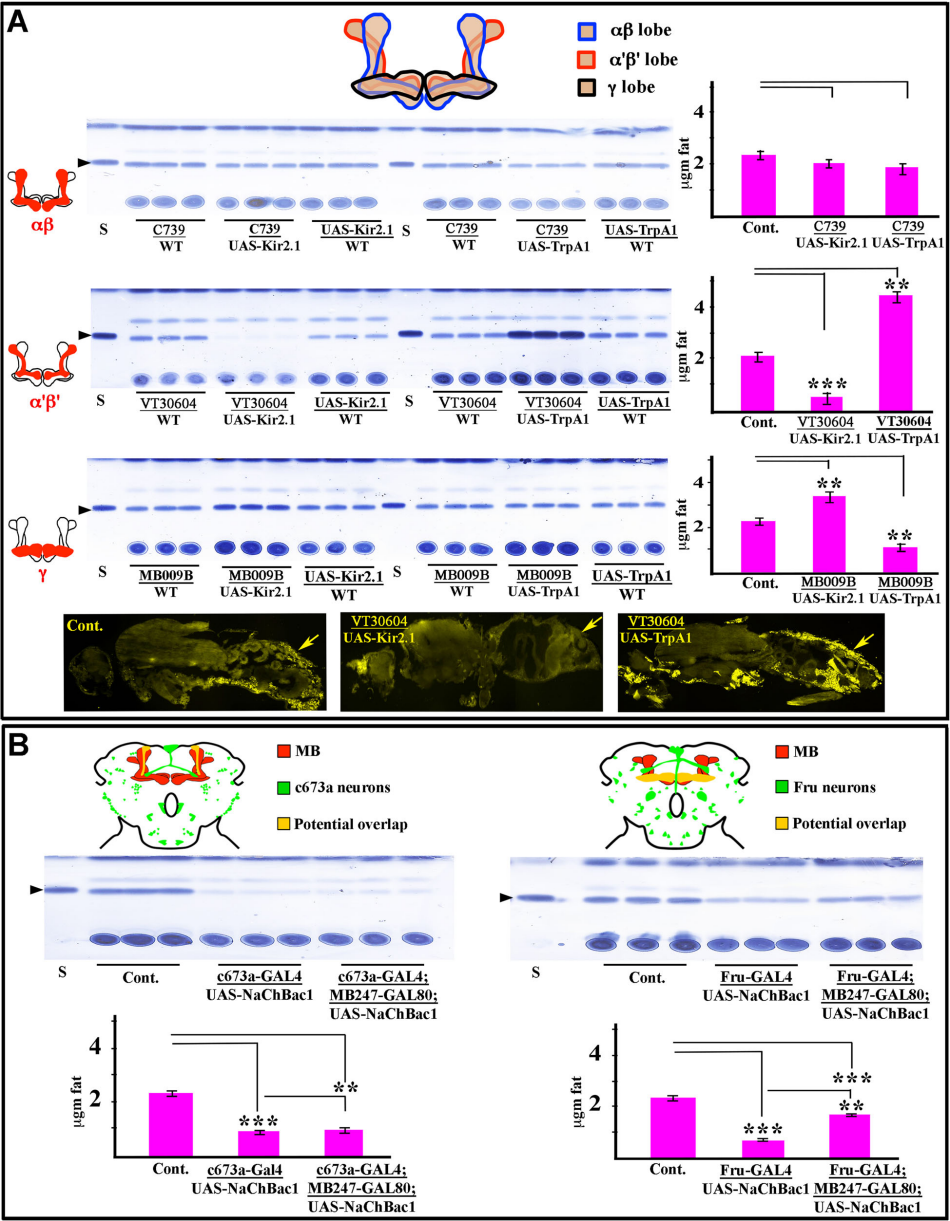
The mushroom body regulates fat storage. **a** Top, cartoon of mushroom body lobes. Left, TLC assays, with 3 replicates for each genotype. For each TLC plate, the left 3 sample sets show Kir2.1-mediated silencing vs. GAL4- and UAS-constructs alone. The right 3 sample sets show dTrpA1-mediated hyperactivation vs. GAL4- and UAS-constructs alone. Right, Quantification of the TLC data. Control bars (Cont.) in the histogram cases are the averages of measurements of four replicas from three different controls (driver/WT, UAS-Kir2.1/WT, and UAS-dTrpA1/WT), all of which have similar fat levels. WT indicates a wild-type chromosome (+). Top row, silencing or hyperactivation of αβ KCs using C739-GAL4 does not affect fat content. Second row, silencing of α’β’ KCs using VT30604-GAL4 produces leanness, while hyperactivation produces obesity. Third row, silencing of γ KCs using MB009-split-GAL4 produces moderate obesity, while hyperactivation produces moderate leanness. Bottom row, the intensity of fat droplet staining by Nile Red is reduced relative to controls when α’β’ KCs are silenced (middle), and increased when they are hyperactivated (right). Arrows indicate fat droplets. **b** Top, schematic illustrations of the fly central brain showing the c673a-GAL4 (left) or Fru-GAL4 (right) neurons in green, MB neurons in red, and overlapping neurons in yellow. Most or all γ KCs express Fru-GAL4. Middle row left, TLC plate showing that hyperactivation of c673a-Gal4-positive neurons using NaChBac1 causes leanness, and silencing GAL4 in all KCs using MB247-GAL80 does not alter the phenotype. Middle row right, TLC plate showing that hyperactivation of Fru-GAL4-positive neurons causes leanness, but silencing GAL4 in all KCs using MB247-GAL80 partially suppresses the effect. Cont. samples for both TLC plates are UAS-NaChBac/+. Bottom row, quantitation of TLC data. S, triglyceride standards. Bars indicate means ± SEM, *n* = 12 samples for pooled controls and *n* = 4 for other genotypes. Asterisks denote t-test statistical significance: **p < 0.005, ***, *p* < 0.0005

The mushroom bodies (MBs) are clusters of neurons in the insect brain that project their axons within tracts resembling pairs of mushrooms. The neurons forming this structure are called Kenyon cells (KCs). In fly, there are 2000–2500 KCs per hemisphere [5, 41]. These cells project axons that form the MB lobes. There are two vertical lobes, α and α’, and three horizontal lobes, β, β’, and γ. αβ KC axons bifurcate and send one branch into the α lobe and one branch into the β lobe, while α’β’ KC axons have one branch in the α’ lobe and one branch in the β’ lobe. Finally, γ KC axons are unbranched and only project to the γ lobe (see diagram in Fig. 1a, top). A large body of evidence demonstrates that the MB plays critical roles in aversive and appetitive learning, sleep, locomotor activity, and decision making [8, 20, 29, 43].

In an earlier study, we developed a fat-specific thin-layer chromatography (TLC) assay [1, 3], and used it to identify two neuronal populations, defined by the c673a and Fru-GAL4 drivers, that cause obesity when silenced and leanness when hyperactivated. Many of the neurons expressing Fru-GAL4 drivers are γ KCs. In this paper, we describe interactions between specific MB neuron activities that regulate fat content in adult flies. We show that KCs forming the α’β’ lobes constitute a new fat regulation center. Perturbation of the activities of KCs that project into the γ lobe produces effects like those previously observed by perturbation of Fru-GAL4 neurons, of which the γ KCs are a subset [3]. We also identified MB input and output neurons that regulate feeding, metabolism, and fat content.

## Methods

### Drosophila strains

Flies were raised as described in [3]. Males from GAL4 lines obtained from various sources and Split-GAL4 lines from Yoshinori Aso at Janelia Farm Research Campus [5, 7] were crossed with females containing either (UAS-Kir2.1, Tub-Gal80^ts^), UAS-dTrpA1, UAS-Shi^ts^, or UAS-NaChBac1. The resulting male progeny were collected for 2 days in groups of 20 individuals. For manipulating neuronal physiology using Kir2.1, dTrpA1, and Shi^ts^, experimental flies and heterozygote controls were shifted to 30 °C for 7–10 days, while NaChBac1-expressing experimental flies and heterozygote controls were incubated at room temperature for 7–10 days before fat content was analyzed. Wild-type flies were of the Canton-S strain.

It has been reported that feeding habits and metabolic demands of female flies changes with mating [10, 38, 46, 49]. To avoid this additional level of complication, our analysis was restricted to male flies isolated within 24 h after eclosion and aged accordingly on standard media.

#### Fat level analysis

Stored fats are composed of a glycerol backbone attached to three fatty acids. These fatty acids are typically not uniform in length or saturation levels. This level of chemical heterogeneity necessitates fat measuring methods that are not targeted to specific species of triglyceride fat but have a broad spectrum. We previously developed a fat detecting thin-layer chromatography (TLC) assay that is superior in terms of specificity and accuracy to the frequently used colorimetric assay [1–3]. Fly extracts analyzed using this assay produce four bands. We previously analyzed the composition of these bands by mass spectrometry. Starting from top (near the solvent front) and going through the bands to the bottom (where the samples were pipetted into the TLC plate), the uppermost band has the migration rate and mass spectromeric pattern of waxes and does not exhibit a change in level during starvation. The second band also does not exhibit a change in level during starvation, but due to its low levels we were unable to identify its identity by mass spectrometry. The third band is triglyceride (fat), and three observations confirm its identity: First, the band has the same migration rate as butter, lard, or a triglyceride standard. Second, it gradually disappears with continued starvation, which is expected of stored fat. Third, analysis by mass spectrometry confirms that this band is composed of a mixture of triglycerides. The final bands (near the base of the TLC assay) appear as two concentric circles and hardly migrate on the TLC assay. Mass spectromeric analysis indicates that they are mixtures of mono- and diglycerides, and they do not show any alteration during starvation [1, 3]. The invariant level and migration pattern of the uppermost bands (waxes and other) does not change when the same number of flies are processed with the same volume of solvent, thus making them useful standards to ensure that the same amount of material was loaded in each lane.

Fly fat extraction and TLC analysis was performed as described by [3]. To quantify fat levels, 1 mg/mL lard standard solution dissolved in 2:1 chloroform:methanol solvent was prepared. When test samples were examined, four different lard standard aliquots were pipetted onto the same TLC plate, providing total lard amounts of 0.5, 1, 2, and 4 micrograms. After staining, the TLC plate was scanned and the average pixel density of each standard sample was measured and used to plot a dose-response curve. The test sample pixel densities were then measured and their fat content determined relative to the trend line slope of the standard samples.

**Fig. 2:**
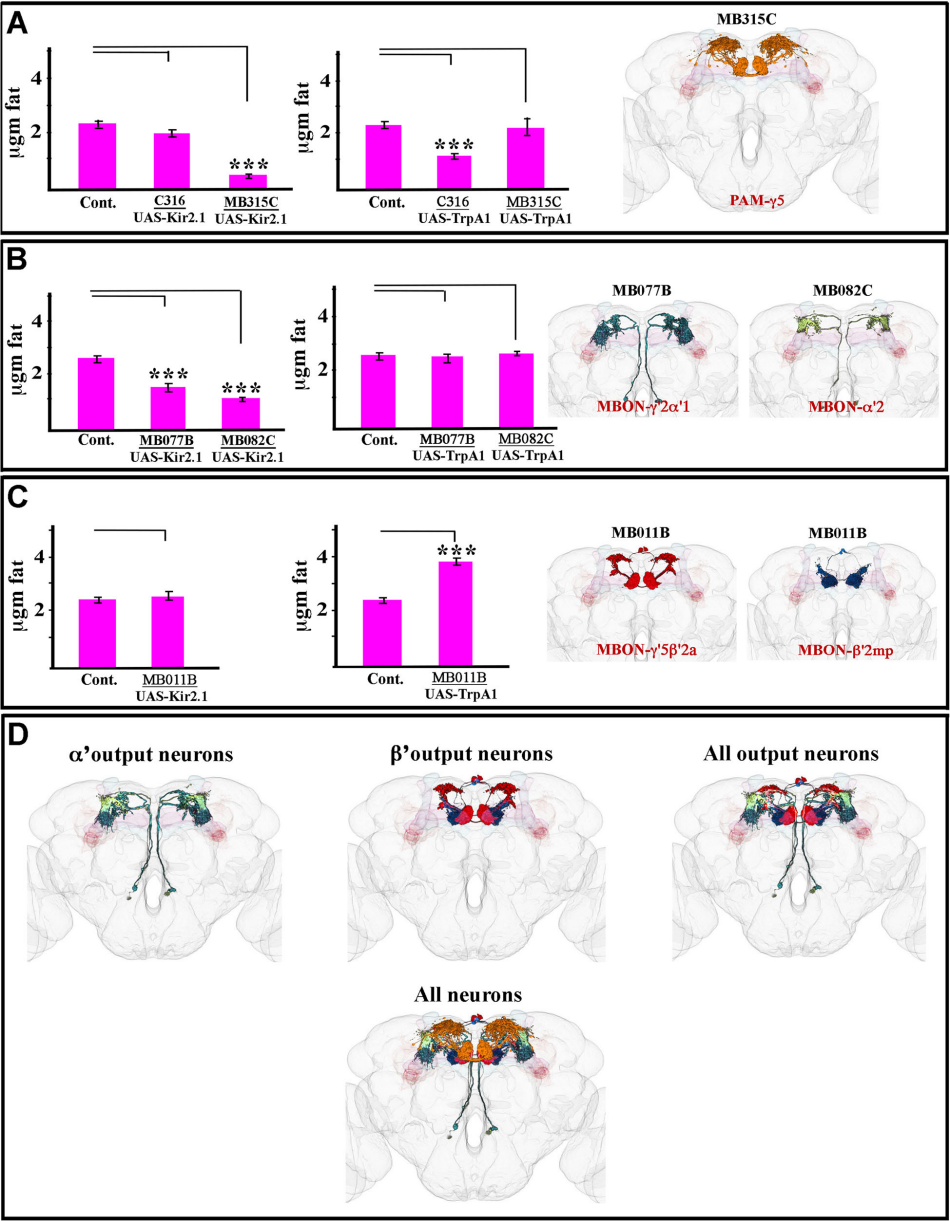
Mushroom body input and output neurons involved in fat storage regulation. **a** Input neurons. Quantitation of fat levels, as measured by TLC, in flies with Kir2.1-mediated silencing (left) or dTrpA1-mediated hyperactivation (right) of DPM neurons using C316-GAL4, or of PAM-γ5 neurons using MB315-GAL4. Far right, Image of PAM-γ5 neurons (orange). In all images in this figure, the brain is a translucent grey skeleton, and the MB lobes are in translucent pink and blue. There are 8–12 PAM-γ5s per brain hemisphere. **b** MBONs innervating α’ compartments. Left bar graph shows that Kir2.1-mediated silencing of MBON-γ2α’1 using MB077B-GAL4 and MBON-α’2 using MB082C-GAL4 causes leanness. Right bar graph shows that dTrpA1-mediated hyperactivation of these neurons has no effect. Far right, images of MBON-γ2α’1 (blue-green) and MBON-α’2 (light green). There are 2 MBON-γ2α’1 s and 1 MBON-α’2, per brain hemisphere. **c** MBONs innervating β’2 compartments. Left bar graph shows that Kir2.1-mediated silencing of MBON-γ5β’2a and MBON-β’2mp neurons using MB011B-GAL4 has no effect on fat content. Right bar graph shows that dTrpA1-mediated hyperactivation of the same neurons causes obesity. Far right, images of MBON-γ5β’2a (red) and MBON-β’2mp (blue). There is 1 MBON-γ5β’2a and 1 MBON-β’2mp per brain hemisphere. **d** Combined images at the bottom show superimpositions of all α’-innervating MBONs (left), all β’-innervating MBONs (middle), all MBONs (right), and MBONs plus PAM-γ5s (bottom). Bars indicate means ± SEM, *n* = 12 samples for pooled controls as in Fig. 2 and *n* = 4 for other genotypes, each composed of 10 flies homogenate. Asterisks denote t-test statistical significance: ***, *p* < 0.0005

To assess the reliability of our data, we compared all pairs of three different genotypes: flies with both driver and transgene lines (test sample), flies with transgene line only (control 1), and flies with driver line only (control 2). Three two-sided independent t-tests were performed: test sample vs. control 1, test sample vs. control 2, and control 1 vs. control 2. Only outcomes in which the mean of the test samples was reliably different from both controls were considered bona fide. Additional file 1: Table S1 presents all comparisons. However, since the controls were all very similar to each other, in the final presentation of the data in the figures we typical show control 1. The exception to this rule was for graphs in which a normal GAL4 and a split-GAL4 line are compared. In these cases (e.g., Fig. 2a), since the GAL4 heterozygotes would be different, we used UAS-Kir2.1/+ or UAS-dTrpA1/+ for the control bars.

#### Behavioral and metabolic assays

Nile red histological staining was performed as described by [1, 3]. Behavioral and metabolic analyses were done using UAS-Kir2.1, UAS-dTrpA1, and UAS-Shi^ts^ animals and controls 2 days after they were shifted to 30 °C. NaChBac1 animals and their controls were incubated at room temperature for 2 days before being examined. The CAFE feeding assay was performed as described by Ja and others and Al-Anzi and others [4, 22]. The climbing assay was performed as described by Nichols and others [31]. Conversion of consumed radiolabeled amino acids into different metabolites was done as described by [3], except that ^14^C leucine was replaced by ^14^C aspartic acid.

Unless stated otherwise, behavioral and metabolic analysis were done on test flies and their controls 2 days after being shifted to 30^o^ C as they were still in the process of either becoming obese or lean.

To assess the reliability of our data, we compared all pairs of three different genotypes: flies with both driver and transgene lines (test sample), flies with driver line only (control 1), and flies with transgene line only (control 2). Three two-sided independent t-tests were performed: test sample vs. control 1, test sample vs. control 2, and control 1 vs. control 2. Only outcomes in which the mean of the test samples was reliably different from both controls were considered bona fide. Additional file 3: Table S3 presents all comparisons. The controls were all very similar to each other, as for the measurement of fat content (see Additional file 1: Table S1). Therefore, in the final presentation of the data, for simplicity we show control 1. The exception to this rule was for graphs in which a normal GAL4 and a split-GAL4 line are compared. In these cases, since the GAL4 heterozygotes would be different, we used UAS-Kir2.1/+ or UAS-dTrpA1/+ for the control bars.

## Results

### The α’β’ and γ lobes of the mushroom body affect fat storage

To determine whether KC activity regulates fat storage, we used MB lobe-specific GAL4 lines to drive the expression of UAS-linked transgenes that cause temperature-dependent conditional neuronal silencing or hyperactivation. To silence neurons, we used an inward rectifier potassium channel, Kir2.1 [9], whose expression is controlled by a ubiquitously expressed temperature-sensitive GAL4 inhibitor, Tub-GAL80^ts^ [34]. To hyperactivate neurons, we used a temperature-activated transient receptor potential cation channel, dTrpA1 [17, 33]. We shifted adult flies bearing MB lobe-specific GAL4 drivers and Kir2.1/GAL80^ts^ or dTrpA1, respectively, to 30 °C for 7–10 days.

This prolonged incubation, while not physiological with respect to neuronal activation, is necessary to allow for fat accumulation, and was also used in earlier studies [3]. As controls, we subjected lines bearing only the DNA constructs for the GAL4 driver or the UAS effectors to the same temperature shifts. This is the appropriate control, because this is not a conditional experiment with respect to temperature. High temperature is necessary to turn on expression of Kir2.1 (through GAL80^ts^ inactivation) and to activate dTrpA1. However, metabolism and fat content change dramatically with temperature, so it would be inappropriate to compare flies at the permissive vs. nonpermissive temperatures. By comparing driver-alone and effector-alone lines to the driver-effector combination at the same temperature, it is possible to accurately assess the effects of the perturbations. In Additional file 1: Table S1, all comparisons to controls are shown. Since the driver-alone and effector-alone lines all have very similar fat contents, in the figures we only show controls 1. Aso and others have generated a large number of split-GAL4 driver lines that label subsets of input mushroom body neurons [7]. The split-GAL4 version they used is based on [35], and is susceptible to GAL80 inhibition.

Silencing or hyperactivation of αβ KCs using C739-GAL4 [5] had no effect on fat content (Fig. 1a, top panels). By contrast, silencing of α’β’ KCs using the VT30604-GAL4 driver [50] produced a dramatic decrease (> 5-fold) in the intensities of the triglyceride bands. Conversely, hyperactivation of α’β’ KCs increased triglyceride content by more than 2-fold (Fig. 1a, second panel). We confirmed that these changes reflected alterations in the numbers of stored fat droplets by Nile Red staining of cryostat sections (Fig. 1a, bottom panel). Two other α’β’ drivers, VT57244-GAL4 [50] and C305a-GAL4 [5], produced similar results (Additional file 1: Table S1). Silencing of γ KCs using the split-GAL4 driver MB009B [7] produced the opposite effect to α’β’ silencing, causing a moderate increase in triglyceride content, while hyperactivation produced a decrease in fat (Fig. 1a, third panel).

Transgenic hyperactivation agents such as cation channels can generate sustained neuronal firing rates that are never observed in normal animals, raising the possibility that what is observed when these agents are used may not be relevant to normal physiology, especially when they are kept on for a prolonged period of time as in our experiments. However, in the case of the two groups of MB neurons described above, we demonstrated that silencing of these neurons generates effects opposite to those produced by hyperactivation. This suggests that the activities of these neurons are indeed relevant to fat storage regulation in normal animals.

As stated in the introduction, in our previous work we identified two fat-regulating neuronal populations, defined by their expression of the c673a-GAL4 and Fru-GAL4 drivers. There is little overlap between the two populations, and the overlapping neurons are not responsible for their phenotypes [3]. However, there is some overlap between MB neurons and neurons expressing c673a-GAL4 and Fru-GAL4 (Fig. 1b, top). In particular, most γ KCs express Fru-GAL4 [3, 18], so the effects caused by silencing and hyperactivation of γ KCs might reflect a role of Fru neurons. To test this, we hyperactivated c673a and Fru neurons with UAS-NaChBac1 (a bacterial cation channel) [32], because dTrpA1-mediated hyperactivation of either of these sets of neurons is lethal. This was done in the presence of a GAL80 repressor element that suppresses GAL4 mediated-expression in the MB. GAL80 expression is driven using a pan-KC promoter element, MB247-GAL80 [42]. MB247-GAL80 had no effect on the leanness phenotype produced by UAS-NaChBac1 hyperactivation of c673a neurons (Fig. 1b, left). However, when we combined Fru-GAL4 and UAS-NaChBac1 with MB247-GAL80, we found that the leanness phenotype was significantly weakened (Fig. 1b, right). This shows that the effect of hyperactivation of Fru neurons on fat content can partially be assigned to γ KCs. However, Fru neurons outside the MB also contribute to fat storage, since flies in which NaChBac1 was shut off in the MB were still significantly leaner than wild-type controls.

### Identification of compartment-specific mushroom body input and output neurons that affect fat storage

The MB receives input from many modulatory neurons, including serotonergic/GABAergic Dorsal Median Paired neurons (DPMs)[19, 47] and dopaminergic neurons called DANs [6, 7, 13–15, 24, 37, 40]. It transmits output signals via a small number of mushroom body output neurons, the MBONs [7]. DAN axons and MBON dendrites have spatially restricted innervation patterns that divide the MB into 15 compartments. Various combinations of compartments regulate aspects of mushroom body function such as appetitive learning, aversive learning, and sleep [8].

We screened the Aso collection of split-GAL4 lines expressing in specific MB neuron subtypes by crossing each of the 68 driver lines with UAS-Kir2.1; Tub-GAL80^ts^ or UAS-dTrpA1, respectively, to silence or hyperactivate each DAN and MBON subtype. To identify specific neurons involved in fat storage regulation, we first screened driver lines to identify those that conferred differences in fat levels when crossed with a perturbing agent relative to controls. Second, driver lines initially scored as hits were verified by retesting for significant differences in fat levels in four independent experiments. Third, in order for us to classify a neuronal type as being involved in fat storage, we required that *all* split-GAL4 driver lines that express in this neuronal type must produce alterations in fat levels. Additional file 2: Table S2 shows results for all split-GAL4 drivers.

The observation that hyperactivation of α’β’ KCs causes obesity suggests that α’β’ KCs could be postsynaptic to inhibitory neurons that are also involved in fat storage regulation. One potential set of inhibitory neurons are the DPMs. Hyperactivation of DPM neurons with C316-GAL4 causes leanness, while silencing them does not affect fat storage (Fig. 2a). This data suggests that DPM neurons inhibit α’β’ KCs and constrain their activity-dependent effect on fat storage.

Of 29 split-GAL4 driver lines specific for input DAN neurons, three lines specific for neurons of the PAM cluster produced significant effects on fat levels when used to drive silencing or hyperactivation agents. For one of these, MB315C-split-GAL4, we identified a small group of DANs, the PAM-γ5 neurons, as responsible for the driver’s effect on fat storage (Additional file 2: Table S2). Silencing of these neurons caused leanness, while hyperactivation had no effect (Fig. 2a). For two other two drivers, MB188B-split-GAL4 and MB087C-split-GAL4, we were unable to identify a single neuronal type that was likely to be responsible for their effects (Additional file 2: Table S2).

Among 34 split-GAL4 lines specific for MBON output neurons, we found six that produced significant effects on fat levels when used to drive silencing or hyperactivating agents. For five of these, we were able to assign these effects to specific compartments and MBONs. MB077B and MB077C-split-GAL4s express in only one MBON type (2 cells per brain hemisphere) called MB-γ2α’1, while MB082C-split-GAL4 expresses in MBON-α’2 and MBON-α3. By examining other drivers (Additional file 2: Table S2), we attributed the effects of MB082C-split-GAL4 to MBON-α’2 (1 cell per brain hemisphere). For both MBON-γ2α’1 and MBON-α’2, silencing produced leanness, while hyperactivation had no effect (Fig. 2b).

The effects of MB011B-split-GAL4 and MB074C-split-GAL4 were attributable to output neurons MBON-β’2mp and MBON-γ5β’2a (1 cell each per brain hemisphere) (Additional file 2: Table S2). For these neurons, hyperactivation produced obesity, while silencing had no effect (Fig. 2c). Images of identified fat-regulating neurons are shown in Fig. 2a-d. For the final driver, MB549C-split-GAL4, silencing caused leanness, but we were unable to identify a single neuronal type to which we could attribute its effects (Additional file 1: Table S1 and Additional file 2: Table S2).

Two other split-GAL4 lines are worth mentioning, MB013B and MB022B. The former label the SIFamide neurons, while the latter labels the octopaminergic neurons OA-VPM3 and OA-VPM4. The SIFamide neurons have been implicated in the translation of hunger signals into feeding behavior [28], while OA-VPM3 and OA-VPM4 are known to be involved in appetitive learning [13]. In the TLC assay, hyperactivation of either neuronal type produced a mild increase in fat levels (Additional file 2: Table S2). However, the *p*-values for the significance of this increase as compared to controls are rather modest (0.04 and 0.05, respectively). Thus, we did not examine these drivers further.

### Behavioral and metabolic mechanisms involved in control of fat storage by the mushroom body

Fat levels are influenced by a variety of factors, including food intake, the rate of de novo fatty acid synthesis, the rate of fat store utilization, and the animals’ physical activity. To evaluate the causes of fat content phenotypes in flies where the activities of specific mushroom body-associated neurons were perturbed, we measured these parameters using behavioral and metabolic assays [3].

Food intake was measured using the capillary feeding assay (CAFE) [4, 22]. The rate of de novo fatty acid synthesis was quantified by feeding flies for 2 days with radioactively labeled amino acids, followed by measuring the amount of radiation incorporated into lipid fractions generated from whole-body extracts [3]. Behavioral activity levels were assessed using a climbing assay [31]. Finally, the rate of fat store utilization was evaluated by measuring the reduction in fat levels due to starvation as described by [3]. Fat was extracted from starved flies in 12 h intervals. The extract was run on the TLC assay, and the fat levels plotted. The slope of the trendline of the fat levels was used to evaluate the rate of fat store depletion as starvation continues. A gentler slope means a slower rate of fat storage depletion.

**Fig. 3:**
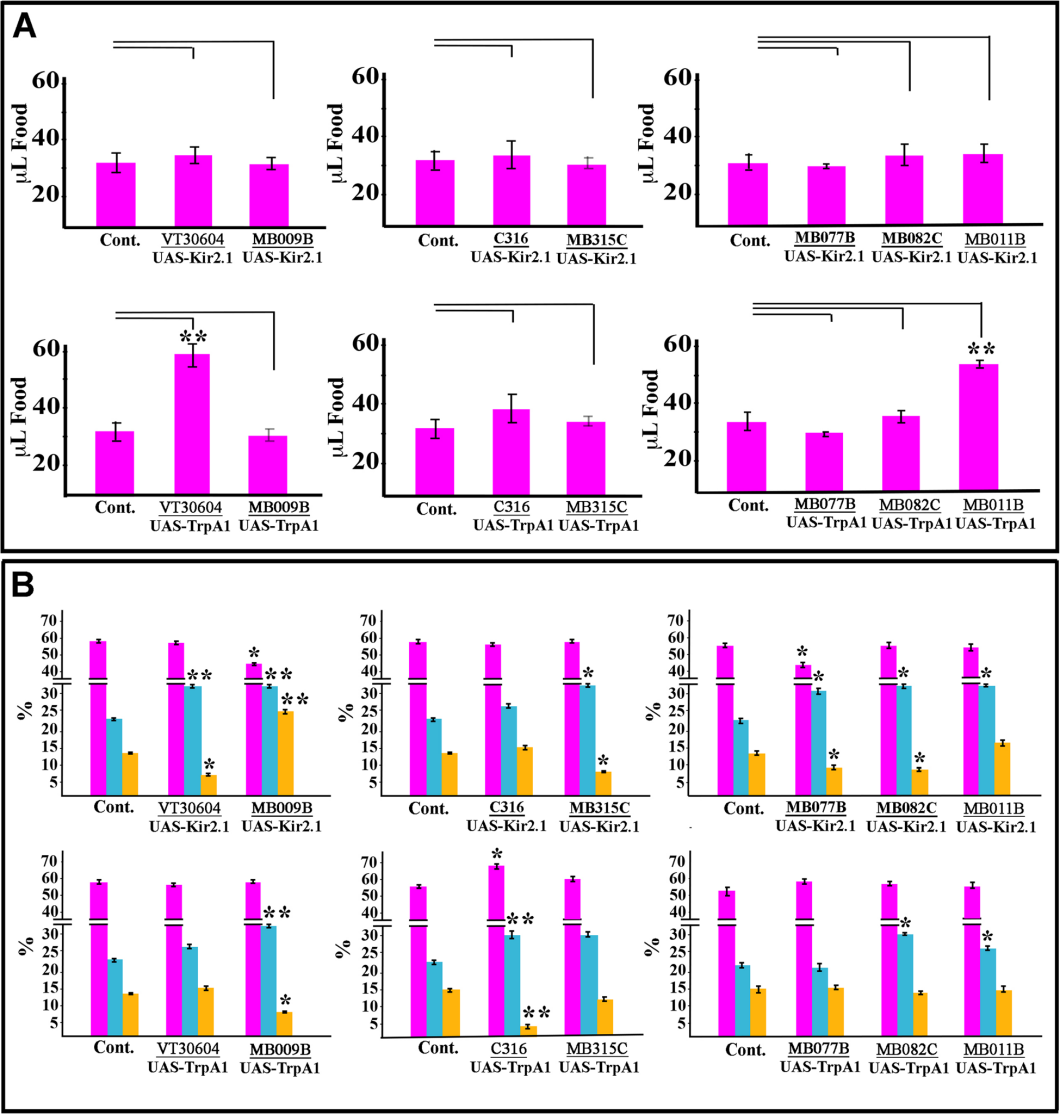
Behavioral and metabolic phenotypes associated with silenced and hyperactivation of the different mushroom body circuits involved in fat storage regulation. **a** Food intake in flies with silenced or hyperactivated MB. Silencing of any type of mushroom body-associated neuron does not significantly affect food intake (upper row). Hyperactivation of α’β’ KCs, using VT30604-GAL4, (lower left) of MBON-γ5β2’a, MBON-β2’mp (MB011B-GAL4 neurons; lower right) cause increases in food intake; all other genotypes are not significantly different from controls. **b** Conversion of ingested ^14^C aspartic acid to protein (magenta), carbohydrate (light blue), and lipid (yellow) in flies with silenced or hyperactivated MB neurons. Silencing of α’β’ KCs (VT30604), MBON-γ2α’1 (MB077B), MBON-α’2 (MB082C), and PAM-γ5 (MB315C) produces decreases in labeled lipids, and silencing γ KCs (MB009B) produces an increase (upper panels). Hyperactivating γ KCs, using MB009-GAL4, and DPM neurons, using C316-GAL4, produces decreases in labeled lipids (lower panels). Bars indicate means ± SEM, *n* = 60 single flies in pooled controls (see Fig. 2) and *n* = 20 for all other genotypes for Café and climbing assay, and *n* = 12 for pooled controls and *n* = 4 for other genotypes for the fat store degradation and ^14^C incorporation experiments (each sample is a homogenate from 10 flies). Asterisks denote t-test statistical significance: *p < 0.05, **p < 0.005, ***, *p* < 0.0005

Hyperactivation of α’β’ KCs, which causes obesity, produced a doubling of food intake (Fig. 3a, lower left), but had no effect on de novo fatty acid synthesis (Fig. 3b, lower left). Silencing of the same neurons, which causes leanness, had no effect on food intake (Fig. 3a, top left), but significantly decreased de novo fatty acid synthesis and increased carbohydrate synthesis (Fig. 3b, top left). For γ KCs, the silencing-induced obesity phenotype was associated with a large increase in de novo fatty acid synthesis and increased carbohydrate synthesis, while hyperactivation-induced leanness was associated with a decrease in de novo fatty acid synthesis, but an increase in carbohydrate synthesis (Fig. 3b, left). Food intake was not significantly affected by either hyperactivating or silencing γ KCs. No abnormalities in climbing rate were detected when α’β’ or γ KCs were silenced or hyperactivated [31]. Additional file 3: Table S3 shows the complete set of comparisons to controls for all of these assays. The results show that the activity of α’β’ KCs affects both food intake and metabolism, while γ KC activity affects only metabolism.

We then evaluated the behavioral and metabolic effects caused by silencing or hyperactivating mushroom body input neurons (DPM and PAM-γ5 neurons) or output neurons (MBON-γ2α’1, MBON-α’2, MBON-β’2mp, and MBON-γ5β’2a). Hyperactivation of DPM input neurons, which causes leanness, produced a decrease in de novo fatty acid synthesis. Silencing PAM-γ5 input neurons, which also causes leanness, produced both a decrease in de novo fatty acid synthesis and an increase in carbohydrate synthesis (Fig. 3b). For output neurons, hyperactivation of MBON-β’2mp and MBON-γ5β’2a (with MB011B), like hyperactivation of their corresponding α’β’ KC inputs, caused obesity and increased food intake but had no effect on de novo fatty acid synthesis (Fig. 3a, b). Silencing MBON-γ2α’1 and MBON-α’2 (with MB077B and MB082C), like silencing α’β’ KCs, caused leanness and produced a decrease in de novo fatty acid synthesis without affecting food intake (Fig. 3a, b). See Additional file 3: Table S3 for the complete dataset.

Hyperactivation and silencing of MBONs involved in fat regulation did not alter climbing rate [31] (Additional file 3: Table S3)*.* These MBONs had previously been assessed for effects on general activity levels, but no major alterations were found when they were silenced or hyperactivated [8, 45].

In summary, we can associate specific MB compartments with behavioral and metabolic effects of perturbation of KCs activity. Increased activity of α’β’ KCs causes an increase in food intake and produces obesity, and these responses match the effects produced by hyperactivating the output neurons MBON-β’2mp and MBON-γ5β’2a, which innervate the β’2 compartment. Decreased activity of α’β’ KCs causes a decrease in de novo fatty acid synthesis and produces leanness, and these responses match those produced by silencing the output neurons MBON-γ2α’1 and MBON-α’2, which innervate the α’1 and α’2 compartments.

### Paired manipulations reveal relationships among fat-regulating neurons and genes

**Fig. 4:**
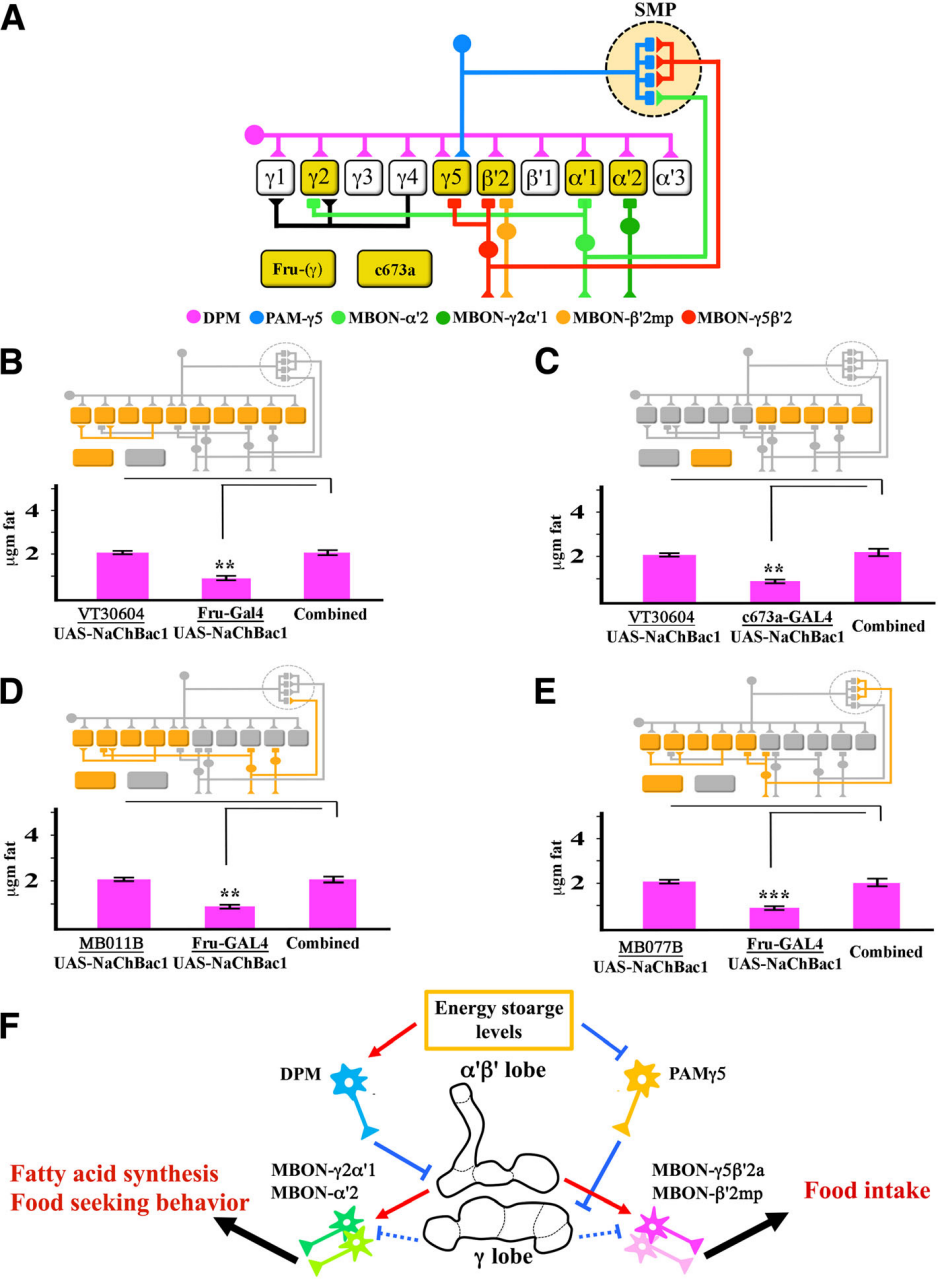
Paired hyperactivation of neurons with opposing effects on fat storage. **a** Schematic illustration of parts of the MB circuitry, including neurons, examined. Solid rectangles are dendritic arbors, and solid triangles are axon terminals. Selected MB compartments in α’β’ and γ lobes are indicated. PAMs and MBONs labeled by the drivers used in this paper are indicated. c673a and non-KC Fru neurons are indicated by unconnected yellow boxes. In the diagrams in the other panels, the method of neural hyperactivation is indicated by red color when dTrpA1 is used and orange color when NaChBac1, highlighted on a grey schematic of the MB circuit. **b** and **c** Combining hyperactivation of α’β’ KCs with VT30604-GAL4 with hyperactivation of Fru-GAL4 neurons (**b**) or of c673a-GAL4 neurons (**c**) results in fat levels that are the same as those in flies in which only α’β’ KCs are hyperactivated, showing the dominant role of α’β’ KC neurons in determining fat content. **d** and **e** Combining hyperactivation of MBON-γ5β’2a and MBON-β’2mp with MB011B (**d**) or of MBON-γ2α’1 with MB077B (**e**) with hyperactivation of Fru-GAL4 neurons results in suppression of the leanness produced by hyperactivation of Fru-GAL4 neurons alone. Bars indicate means ± SEM, *n* = 12 samples for pooled controls and *n* = 4 for other genotypes, as in Fig. 1. Asterisks denote t-test statistical significance: *p < 0.05, **p < 0.005, ***, *p* < 0.0005. In all experiments, the combined column refers to a single copy of both drivers (GAL4, and split-GAL4) present with a single copy of UAS-NaChBac1 **f**. A model for fat-regulating MB circuitry. α’β’ KCs regulate both food consumption (via MBONs innervating β’ compartments) and fatty acid synthesis (via MBONs innervating α’ compartments). MBON-γ5β’2a and MBON-γ2α’1 also innervate the γ lobes. γ KC activity affects fatty acid synthesis but not food consumption. The phenotypes shown in Fig. 3 and the epistatic relationships shown in Fig. 4 suggest that the output of the γ KCs that is relevant to fat storage is opposite in sign to that of the α’β’ KCs. However, we do not know the circuits through which γ KCs regulate fat content, so connections from the γ lobes are shown as dotted lines with inhibition bars

We next examined the functional ordering of pairs of different fat-storage-regulating neurons that (a) either partially or fully overlap with the mushroom body, and (b) have an opposite effect on fat storage when activated. This may provide insights into the functional ordering of these elements. The drivers examined include c673a-GAL4 and Fru-GAL4, which were identified in our earlier study using NaChBac and Shi^ts^ [3], and the drivers described in this paper for α’β’ KCs, MBON-β’2mp, MBON-γ5β’2a, and MBON-γ2α’1 (see Fig. 4a for diagram of circuitry).

We first examined interactions between α’β’ KCs and the previously identified c673a-GAL4 and Fru-GAL4 neurons [3]; Fru-GAL4 neurons include γ KCs. Hyperactivation with dTrpA1 is lethal with c673a-GAL4 and Fru-GAL4. Since flies can survive without MB (Sweeney et al., 2012), this observed lethality is probably due to Fru-GAL4 and c673a-GAL4 expression in neuronal population outside the MB. In order to examine the combined manipulations of Fru-GAL4 or c673a-GAL4 with different MB drivers, we had to use neuronal hyperactivation mediated by UAS-NaChBac1 that does not cause such lethality (Al-Anzi et al., 2009). However, we observed that UAS-NaChBac1 does not produce effects on fat content when expressed in MB or MB associated neurons (Figure 4), suggesting that the increased activity in these neurons when induced by UAS-NaChBac1 is not sufficient to alter fat storage. However, since we know that these transgenes do cause changes in fat content when used with c673a-GAL4 or Fru-GAL4, we infer that UAS-NaChBac1 is likely to produce an alteration in the activity of MB neurons. In fact, we observed that α’β’ KC hyperactivation with UAS-NaChBac1 completely suppressed leanness produced by hyperactivation of c673a or Fru neurons (Fig. 4b, c), confirming that output from α’β’ neurons is the most important controller of fat content. In a similar manner, we asked whether the effects of hyperactivation of Fru neurons might be modulated by MBONs that receive input from both α’β’ and γ lobes. To do this, we hyperactivated MBON-γ5β’2a and MBON-β’2mp (MB011B neurons), and MBON-γ2α’1 (MB077B) output neurons, together with Fru neurons. We observed that hyperactivation of either MBON-γ5β’2a and MBON-β’2mp, or of MBON-γ2α’1, suppressed the leanness phenotype conferred by Fru neuron hyperactivation (Fig. 4d, e). Note that in all four of these cases the MB driver suppresses the effect of the Fru or c673a driver. We can infer that the Fru and c673a drivers are stronger than the MB drivers since they produce lethality when used to drive dTrpA1, while the MB drivers do not. Thus, the effect of the combined drivers is unlikely to be due to dominance by the stronger GAL4 driver.

## Discussion

In this paper, we demonstrate that the mushroom body plays a central role in regulation of fat levels in *Drosophila*. Hyperactivation of α’β’ KCs causes obesity, while silencing causing leanness. Hyperactivation and silencing of γ KCs produces the opposite effects (Fig. 1). α’β’ KCs are a fat storage regulating center that dominates the fly’s physiology, while γ KCs are a subset of the Fru-GAL4 neurons previously implicated in fat store regulation [3]. We also identified MB input and output neurons that mediate these effects.

By combining anatomical analysis and metabolic and behavioral assays, we showed that feeding and fat metabolism are controlled by separate MB output channels (Figs. 2 and 3). Cholinergic MBON-β’2mp and MBON-γ5β’2a (MB011B) output neurons, which innervate the β’2 compartment, regulate food consumption. Glutamatergic MBON-γ2α’1 and MBON-α’2 (MB077B and MB082C) output neurons, which innervate α’ compartments, control fat metabolism.

Some of the neurons we found to be involved in fat storage regulation have been previously reported to have functions in food-related behaviors. PAM neurons that innervate the β’2 and γ5 compartments, including PAM-γ5, have been shown to be involved in appetitive learning [21, 51]. DPM neurons are involved in caloric frustration memory, in which flies learn to avoid non-nutritional sweeteners [30]. MBON-γ2α’1, MBON-α’2, MBON-β’2mp, and MBON-γ5β’2a are needed for both visual and odor-associated appetite learning [8]. MBON-γ2α’1 is required for the acquisition, consolidation, and retrieval of appetitive learning [52].

Recently, Tsao and others have shown that inhibiting the activity of KCs increases the time required for hungry flies to find yeast food after starvation. They identified five MBON pathways for which silencing also impairs food-seeking behavior. These included MBON-γ2α’1 and MBON-α’2, which we identified as important for fat regulation in this paper. Silencing of either MBON-γ2α’1 and MBON-α’2 causes leanness (Fig. 2b). They also reported that these neurons exhibited yeast odor-evoked calcium transients that are modulated by starvation [45]. We did not observe that flies in which MBON-γ2α’1 and MBON-α’2 are silenced consumed less food than controls (Fig. 3a). This does not contradict the findings of Tsao et al., however, because the CAFÉ assay we used does not measure food-seeking. In this assay, the flies are continually exposed to food and the amount of food consumed is measured.

It is interesting that both silencing and hyperactivation of α’β’ KCs produced changes in fat content, while the MBONs we identified as involved in fat regulation segregated into those for which only silencing affects fat content (MBON-γ2α’1 and MBON-α’2) (MB077B and MB982C) and those for which only hyperactivation affects fat content (MBON-β’2mp and MBON-γ5β’2a) (MB011B). This suggests that outputs from α’β’ KCs are compartmentalized, so that the effects of high activity go through a set of cholinergic output MBONs, while the effects of low activity go through a set of glutamatergic MBONs.

Our results, along with previously analyses, are consistent with a model in which the fly uses the activities of α’β’ KCs to maintain fat content within normal levels. A decrease in energy stores might cause a reduction in inhibitory input to α’β’ KCs by decreasing GABA release from DPM neurons. As a consequence of reduction in inhibition by DPM neurons, the activity of α’β’ KCs would increase, resulting in increased transmitter release from α’1, α’2, and β’2 compartments onto the MBON-γ2α’1, MBON-α’2, MBON-β’2mp, and MBON-γ5β’2a neurons. This will prevent the inhibition of MBON-γ2α’1 and MBON-α’2, leading to an increase in food seeking behavior [45], and may also cause an increase in the activities of MBON-β’2mp and MBON-γ5β’2a. This would in turn increase food consumption, leading to a restoration of energy stores (see diagram in Fig. 4f). Conversely, when energy stores are sufficient, DPM inhibits α’β’ activity, resulting in decreased transmitter release from the α’1, α’2, and β’2 compartment onto the above stated MBONs, reducing their activity. This will case a reduction in food seeking behavior and may prevent the release of humoral factors controlling lipid synthesis via MBON-γ2α’1 and MBON-α’2, thus reducing the rate of fat droplet production.

The mechanisms by which the γ KCs influence fat storage are less clear. PAM-γ5 silencing causes leanness and decreased lipid synthesis, like hyperactivation of γ KCs. γ KCs may affect the activities of the MBONs we identified as regulators of lipid synthesis, or act through other MBONs that were not defined by our studies, since the effects of some split-GAL4-mediated perturbations could not be assigned to specific cells (Fig. 4f).

In terms of fat storage regulation, two potential paths of cross-communication between α’β’ KCs and γ KCs are histologically feasible. The first one involves the superior medial protocerebrum (SMP), a brain area in which PAM-γ5 input neuron dendrites are contacted by axonal termini of the output neurons identified by our study [7]. The other is the dendritic arbors of MBON-γ2α’1 and MBON-γ5β’2a, which contact both the α’β’ and γ lobe, potentially receiving input from both simultaneously [7].

## Conclusions

We identified a new fat storage regulating center in the *Drosophila* brain, the α’β’ KCs, which project into the α’β’ lobes of MB. We identified MB input and output neurons involved in its control of fat content. Our findings show that food intake and fat metabolism are controlled by separate sets of postsynaptic neurons that are segregated into different output pathways.

## Additional files

Additional file 1: **Table S1.** Fat level quantifications for fly strains producing statistically significant effects. These are data that are not included in the main figures. (JPG 785 kb)

Additional file 2: **Table S2.** Summary of all split-GAL4 line results. GAL4 line numbers are indicated on the left. The top labels indicate MB neurons. The black and grey squares indicate whether the driver is expressed in that neuron, with darker shades representing stronger expression. These data are from Aso and colleagues. The colors represent our analysis, with red rows denoting hits: drivers that produced consistent, statistically significant effects on fat content when used to drive silencing or hyperactivating agents. Yellow rows indicate drivers for which we observed alterations in fat content, but these alterations were small or the effects were not highly reproducible. Blue rows indicate drivers for which no effects on fat content with silencing or hyperactivation were observed. To assign a neuron as relevant for fat storage, we required that all drivers expressed in that neuron have some effect on fat content, and that at least one of these should be classified as red. The nature of the effect produced by the driver is indicated at the right. This table is based on Additional file 1 in Aso and others [8]. (PNG 121 kb)

Additional file 3: **Table S3.** Summary of CAFÉ assays, climbing assays, fat store degradation, and conversion of ^14^C-labeled-aspartate to different macro-molecular classes. These are data that are not included in the main figures. (JPG 1336 kb)

## Abbreviations

CAFÉ: Capillary assisted feeding assay
DANs: Dopaminergic neurons
DPMs: Dorsal Median Paired neurons
KC: Kenyan cell
Kir2.1: Inward rectifier potassium channel
MB: Mushroom body
MBONs: Mushroom body output neurons
NaChBac: Bacterial cation channel
Shi^ts^: Temperature sensitive shibire
TLC: Thin-layer chromatography
TrpA1: Temperature-activated transient receptor potential cation channel
