# Supplementary figures and images for: Identification and characterization of mushroom body neurons that regulate fat storage in Drosophila
Source: Neural Dev. 2018 Aug 13;13:18. doi: 10.1186/s13064-018-0116-7 (PMC6090720; doi:10.1186/s13064-018-0116-7)

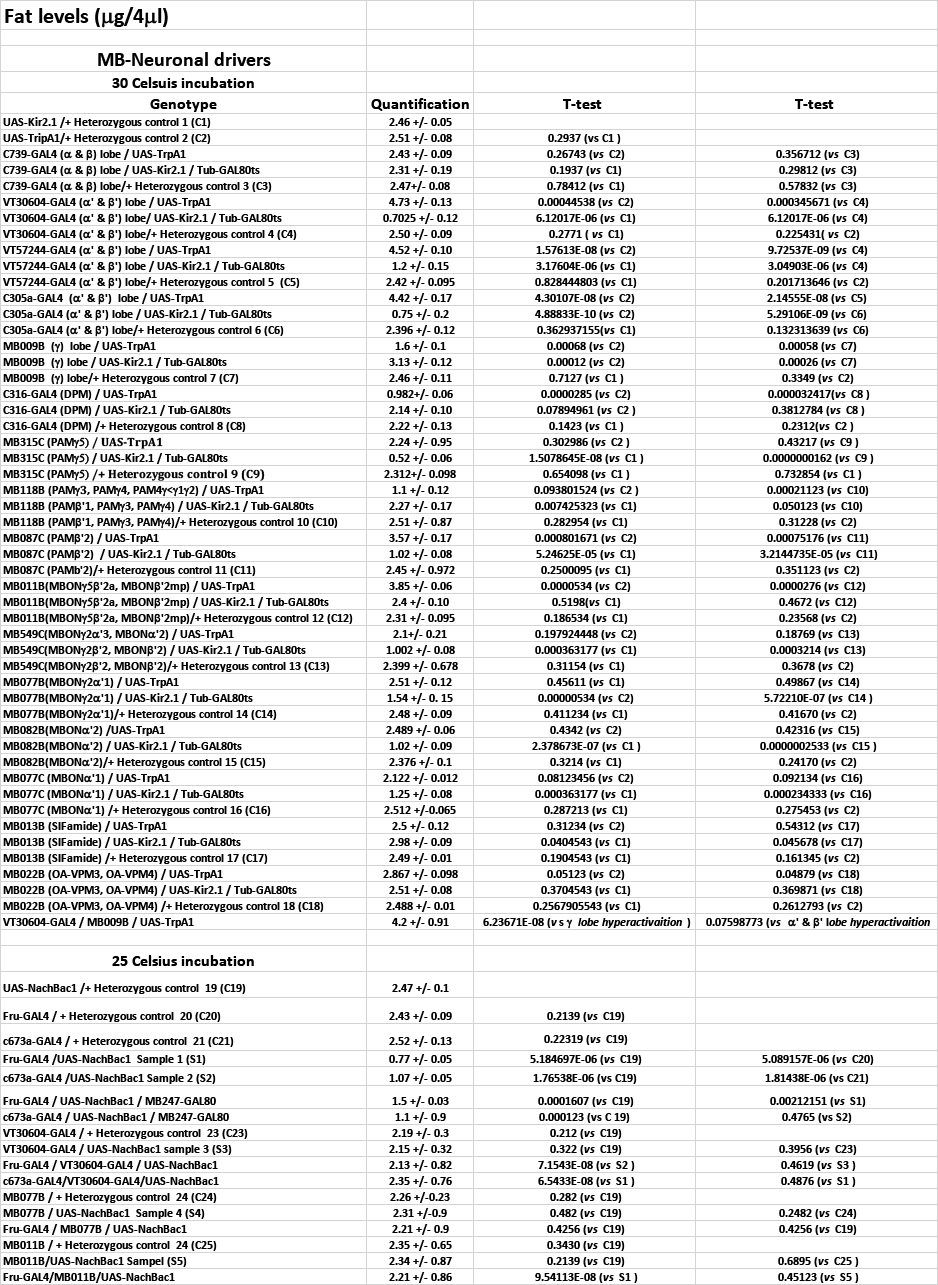

Supplement: Supplementary file 1 — Table S1. Fat level quantifications for fly strains producing statistically significant effects. These are data that are not included in the main figures. (JPG 785 kb) [file 13064_2018_116_MOESM1_ESM.jpg]

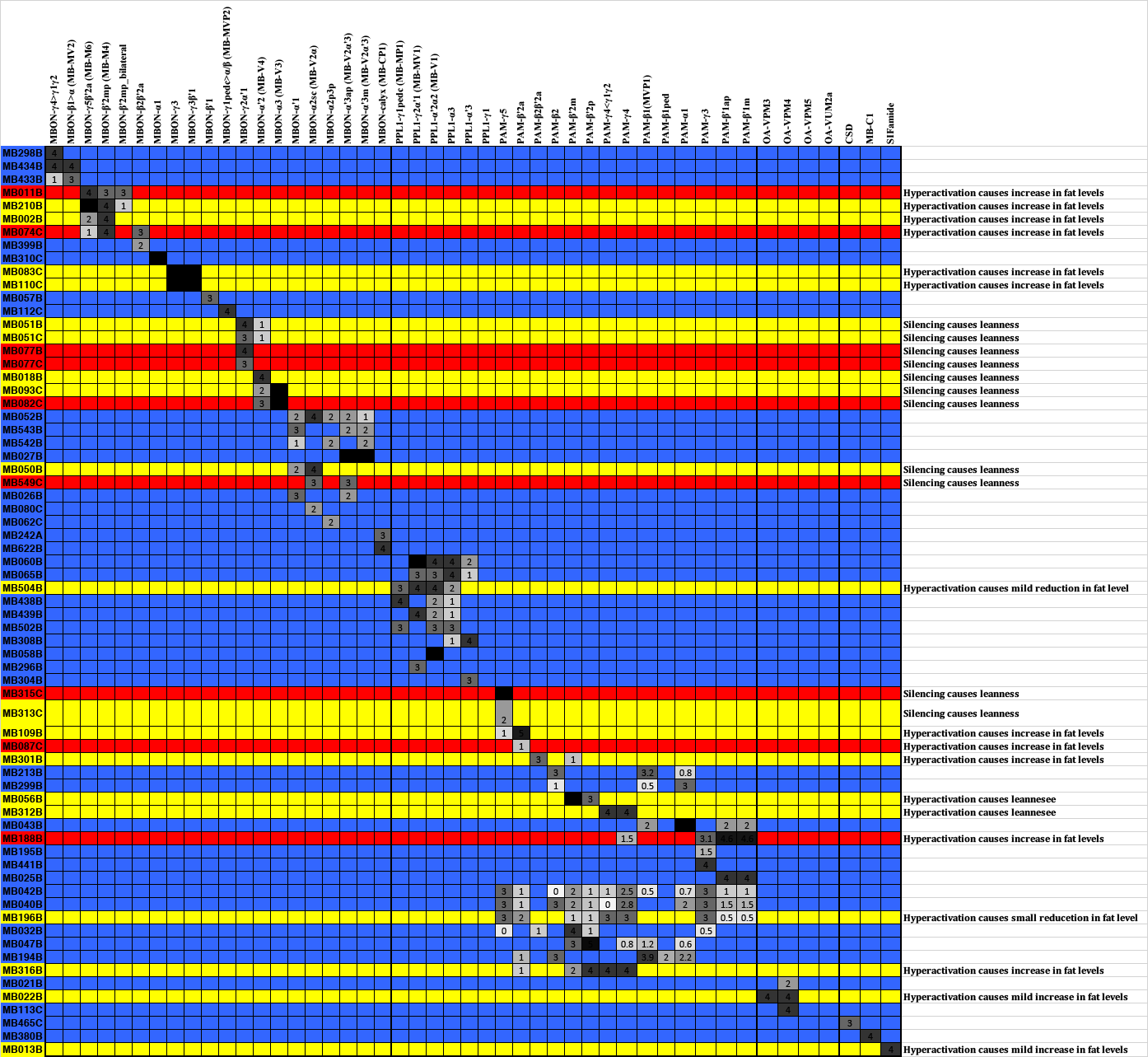

Supplement: Supplementary file 2 — Table S2. Summary of all split-GAL4 line results. GAL4 line numbers are indicated on the left. The top labels indicate MB neurons. The black and grey squares indicate whether the driver is expressed in that neuron, with darker shades representing stronger expression. These data are from Aso and colleagues. The colors represent our analysis, with red rows denoting hits: drivers that produced consistent, statistically significant effects on fat content when used to drive silencing or hyperactivating agents. Yellow rows indicate drivers for which we observed alterations in fat content, but these alterations were small or the effects were not highly reproducible. Blue rows indicate drivers for which no effects on fat content with silencing or hyperactivation were observed. To assign a neuron as relevant for fat storage, we required that all drivers expressed in that neuron have some effect on fat content, and that at least one of these should be classified as red. The nature of the effect produced by the driver is indicated at the right. This table is based on Additional file 1 in Aso and others [8]. (PNG 121 kb) [file 13064_2018_116_MOESM2_ESM.png]

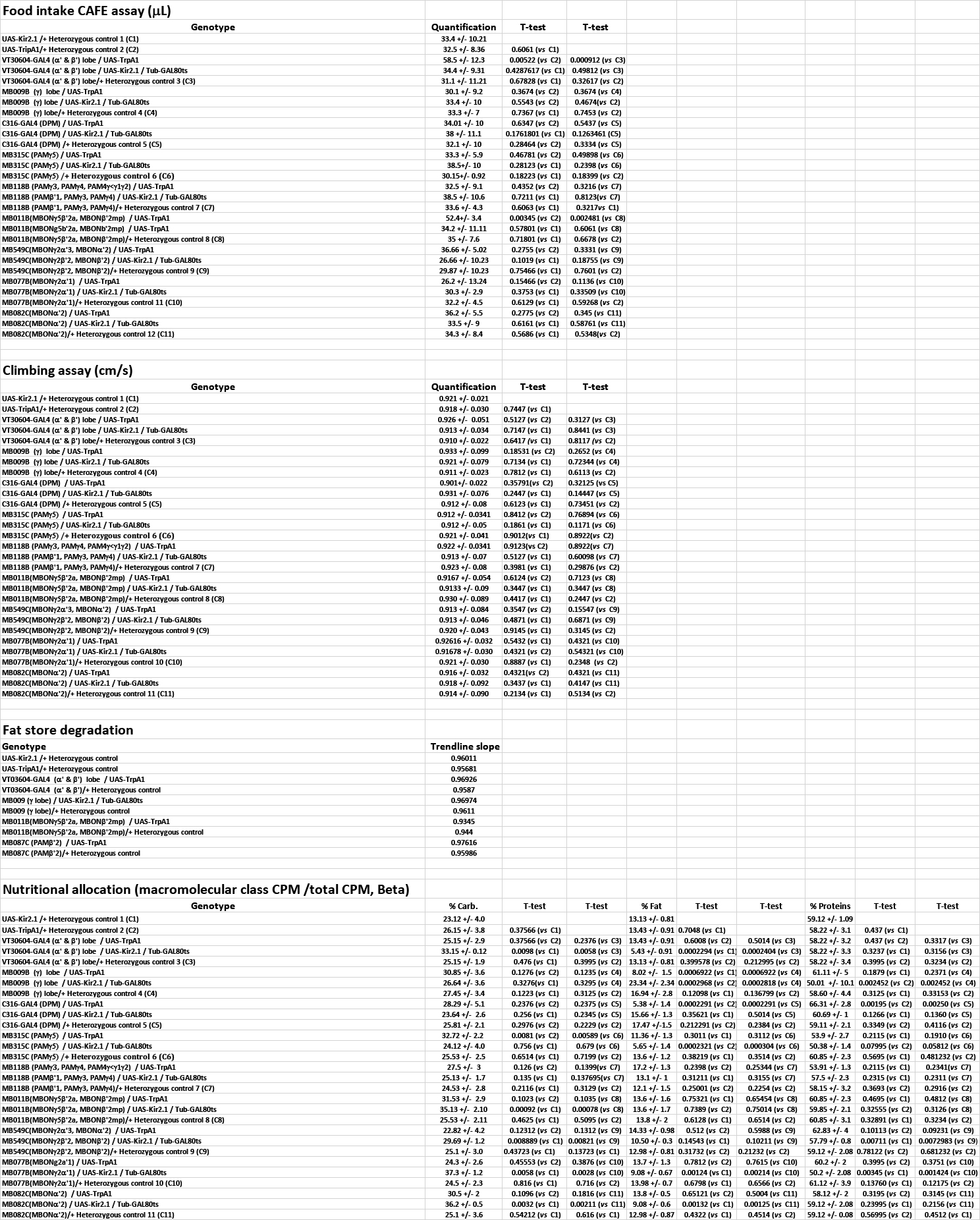

Supplement: Supplementary file 3 — Table S3. Summary of CAFÉ assays, climbing assays, fat store degradation, and conversion of 14C-labeled-aspartate to different macro-molecular classes. These are data that are not included in the main figures. (JPG 1336 kb) [file 13064_2018_116_MOESM3_ESM.jpg]
